# Liver-enriched Genes are Associated with the Prognosis of Patients with Hepatocellular Carcinoma

**DOI:** 10.1038/s41598-018-29237-5

**Published:** 2018-07-25

**Authors:** Binghua Li, Tiancheng Xu, Chaohui Liu, Gang Meng, Yuchen Sun, Liyuan Qian, Shaohe Wang, Jiwu Wei, Decai Yu, Yitao Ding

**Affiliations:** 1 Department of Hepatobiliary Surgery, the Affiliated Drum Tower Hospital, Medical School of Nanjing University, Nanjing, 210093 China; 2 Jiangsu Key Laboratory of Molecular Medicine, Medical School of Nanjing University, Nanjing, 210093 China

## Abstract

Tissue-enriched genes are highly expressed in one particular tissue type and represent distinct physiological processes. The dynamic profile of tissue-enriched genes during tumorigenesis and progression remains largely unstudied. Here, we identified tissue-enriched genes from 12 tissue types based on RNA sequencing data from the Cancer Genome Atlas (TCGA), and found that the liver had the largest number of such genes among the 12 tissue types. The characteristics of liver-enriched genes were further investigated. Most liver-enriched genes were downregulated and metabolism-related genes, which were associated with pathological stage and dedifferentiation in patients with hepatocellular carcinoma (HCC). Hypermethylation might be a mechanism underlying the downregulation of liver-enriched genes. We constructed a liver-enriched gene set and demonstrated that it is associated with the prognosis of the patients with HCC both in the TCGA cohort and the Gene Expression Omnibus (GEO) datasets. Moreover, we discovered that the degree of the dissimilarity between tumors and normal tissues was correlated with the prognosis of patients with HCC and the biological behaviours of the tumors. These results will help identify prognostic biomarkers of patients with HCC, and enhance our understanding of the molecular mechanisms of hepatocarcinogenesis and progression.

## Introduction

The expression of most genes varies between different tissue types and between different physiological and pathophysiological conditions^1,2^. Tissue-enriched genes (TEGs) are highly expressed in one particular tissue type and are either not expressed or are expressed at much lower levels in other tissue types and are generally responsible for the specialized functions of the particular tissues^3^. It is commonly believed that the development and progression of cancers is accompanied by complex changes in the patterns of gene expression^4^. Thousands of genes may be differentially expressed in cancer cells^5^. In recent studies, we discovered that GLS2^6^ and UPB1^7^, two genes previously reported to be predominantly expressed in the liver^8,9^, are downregulated in tumors and are positively correlated with the survival time of patients with HCC. Compared to a single gene analysis, which focuses on identifying differentially expressed individual genes, characterization of the gene expression profile provides comprehensive insight into the molecular events underlying cancer and may help develop better methods for diagnosis or prognosis evaluation of cancer. However, the dynamic profile of TEGs during tumorigenesis and progression has not been elucidated.

With advances in the application and integration of high-throughput screening technology, the challenge has no longer laid in obtaining gene expression profiles, but rather in interpreting the results to gain insight into the biological mechanisms. Gene set enrichment analysis is an approach for interpreting genome-wide expression profiles^10^. However, no tissue-enriched gene set is available in the Molecular Signatures Database (MSigDB).

Hepatocellular carcinoma (HCC) accounts for >90% of all malignant liver tumors and it carries a poor prognosis^11^ with a 5-year survival rate for patients with localized HCC of 30.5%, and <5% for those with distant metastases, according to the Surveillance, Epidemiology, and End Results (SEER) database^12,13^. In contrast to the overall declining trends in the death rates of other major cancers, death rates of liver cancer increased from 2010 to 2014^14^. Thus, it is necessary to identify patients with poor clinical outcomes and adopt effective interventions to improve the prognosis of patients with HCC^15^.

In this study, we systematically profiled TEGs from 12 tissue types using RNA sequencing data from the Cancer Genome Atlas (TCGA)^16^. The results revealed the important role of the liver in TEGs. Then, we analyzed the characteristics of the liver-enriched genes (LEGs) and determined that hypermethylation might partly explain the downregulation of LEGs. We constructed an LEG set that was associated with the prognosis of patients with HCC and demonstrated that the divergence between tumors and normal tissues was related to the prognosis of patients with HCC and the biological behaviours of the tumors. These results offer some integrative insight into the understanding the molecular mechanism of HCC development and its progression, and might contribute to screen prognostic biomarkers in patients with HCC.

## Results

### Pipeline summarizing the integrative approach utilized in this study

**Figure 1:**
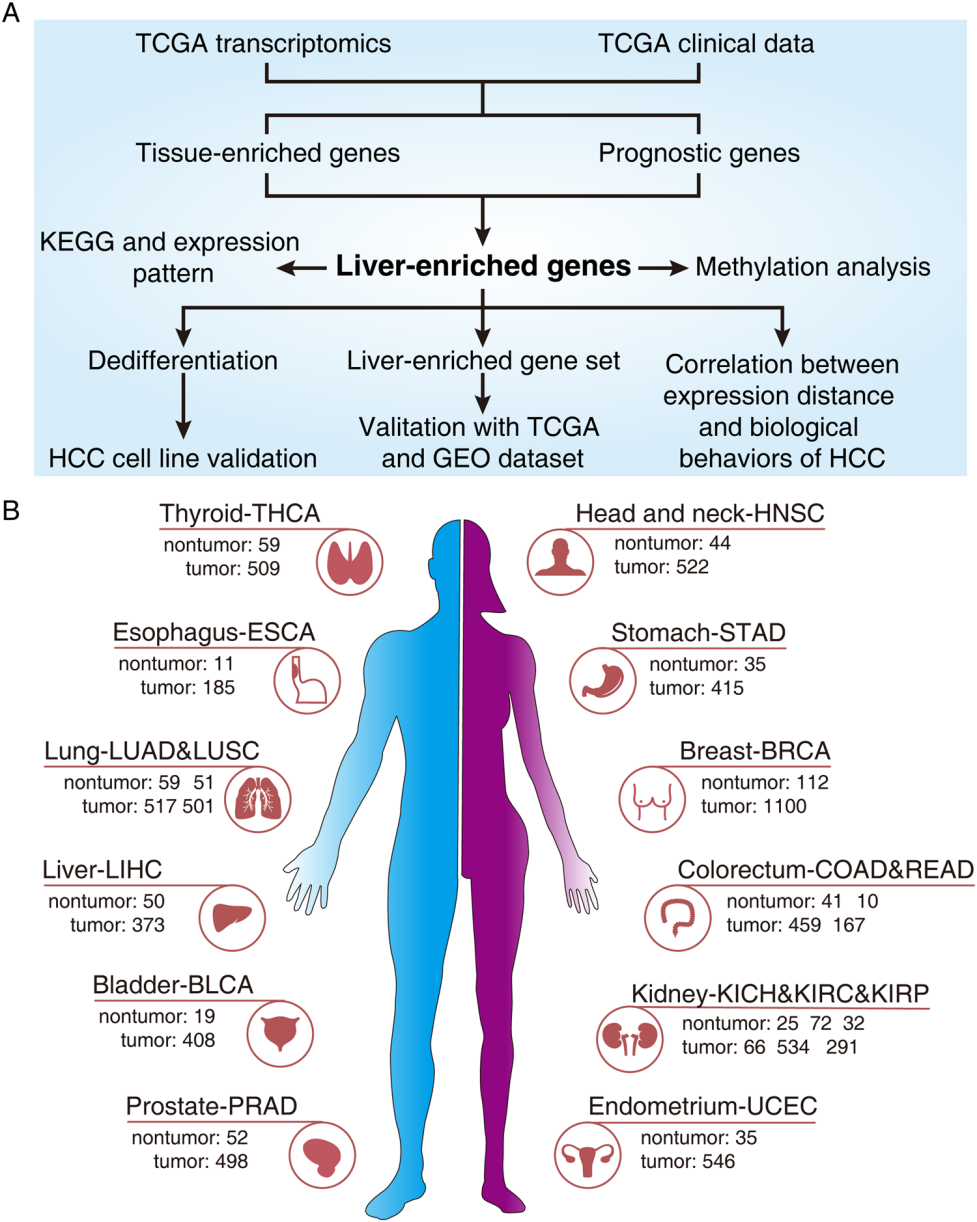
Analysis of the tissue-enriched genes in human cancers. (**A**) Schematic drawing describing the pipeline of the study. (**B**) Tissue and cancer categories included in the study and the corresponding sample size. Cancer types with <10 matching non-tumor samples were ruled out. As a result, 12 tissue types and 16 tumor types were selected, including 707 non-tumor samples and 7,091 tumor samples. Abbreviations: BLCA, bladder urothelial carcinoma. BRCA, breast invasive carcinoma. COAD, colon adenocarcinoma. ESCA, esophageal carcinoma. HNSC, head and neck squamous cell carcinoma. KICH, kidney chromophobe. KIRC, kidney renal clear cell carcinoma. KIRP, kidney renal papillary cell carcinoma. LIHC, liver hepatocellular carcinoma. LUAD, lung adenocarcinoma. LUSC, lung squamous cell carcinoma. PRAD, prostate adenocarcinoma. READ, rectum adenocarcinoma. STAD, stomach adenocarcinoma. THCA, thyroid carcinoma. UCEC, uterine corpus endometrial carcinoma.

An analysis pipeline is shown in Fig. 1A. Briefly, transcriptomics and clinical data were retrieved from the TCGA. Non-tumor samples were used to screen TEGs and tumor tissues were adopted to identify prognostic genes in a variety of tumors. Only cancer categories with more than 10 matching non-tumor samples were included. As a result, 12 tissue types and 16 tumor types were selected, including 707 non-tumor samples and 7,091 tumor samples. The sample sizes of each tissue and cancer types are displayed in Fig. 1B. Given that the LEGs were the greatest in number, and most LEGs were prognostic, the characteristics of the LEGs were further investigated.

### The liver has the largest number of TEGs and most LEGs are prognostic

**Figure 2:**
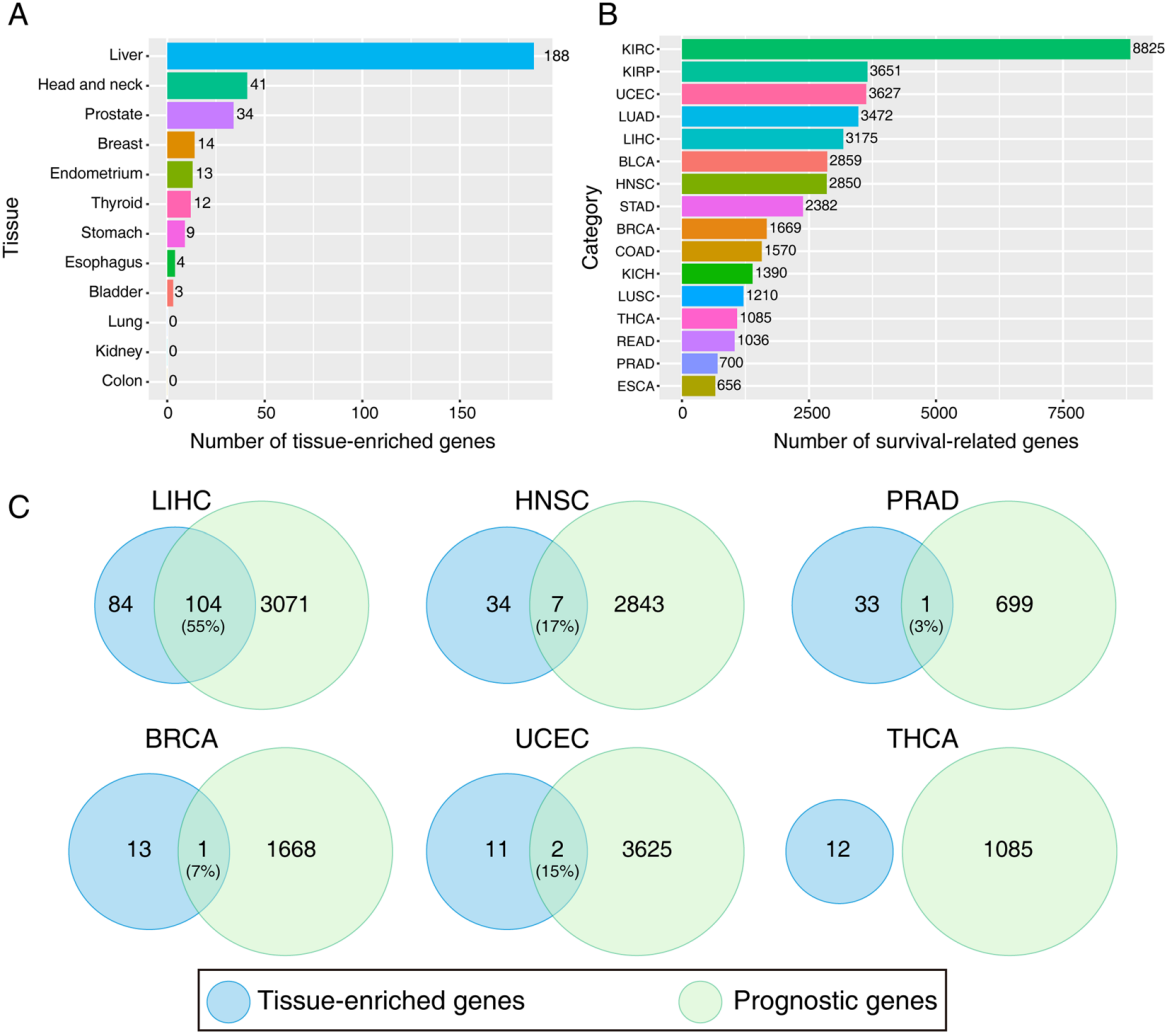
Identification of tissue-enriched genes and prognostic genes. (**A**) Numbers of tissue-enriched genes in the 12 non-tumor tissue types. (**B**) Numbers of survival-related genes in the 16 cancer types. Genes with statistically significant differences (p < 0.05) by the Log-rank test were considered as survival-related genes with a median as cut-off value. (**C**) Venn diagrams showing number of tissue-enriched genes and prognostic genes and their overlap in different cancer types. The percentage of prognostic genes in liver-enriched genes is shown in parentheses.

We screened TEGs from 12 tissue types with the criteria illustrated in the Materials and Methods and identified 188 LEGs, which was much larger than in any other tissue types. In all, 41 and 34 head and neck-enriched and prostate-enriched genes were detected, respectively, yet no TEGs were obtained from the lungs, kidneys, or colon (Fig. 2A, Supplementary Table S1). In addition, we performed survival analysis of every gene in the 16 cancer types in search of prognostic genes (Fig. 2B). A large number of survival-related genes was detected in kidney tumors, particularly in kidney renal clear cell carcinoma (KIRC), which makes up to half of the genomic protein-coding genes. In HCC, 3,175 (approximately 15%) genes were identified as survival-related genes (log-rank, p < 0.05; Fig. 2B).

Then, we overlapped the tissue-enriched and prognostic genes across cancer types. In HCC, 55% (84/188) of the LEGs were prognostic, which was much higher than the average percentage (about 15%), while the proportion was much lower in the other cancer types (Fig. 2C).

### Most LEGs are metabolism-associated genes

**Figure 3:**
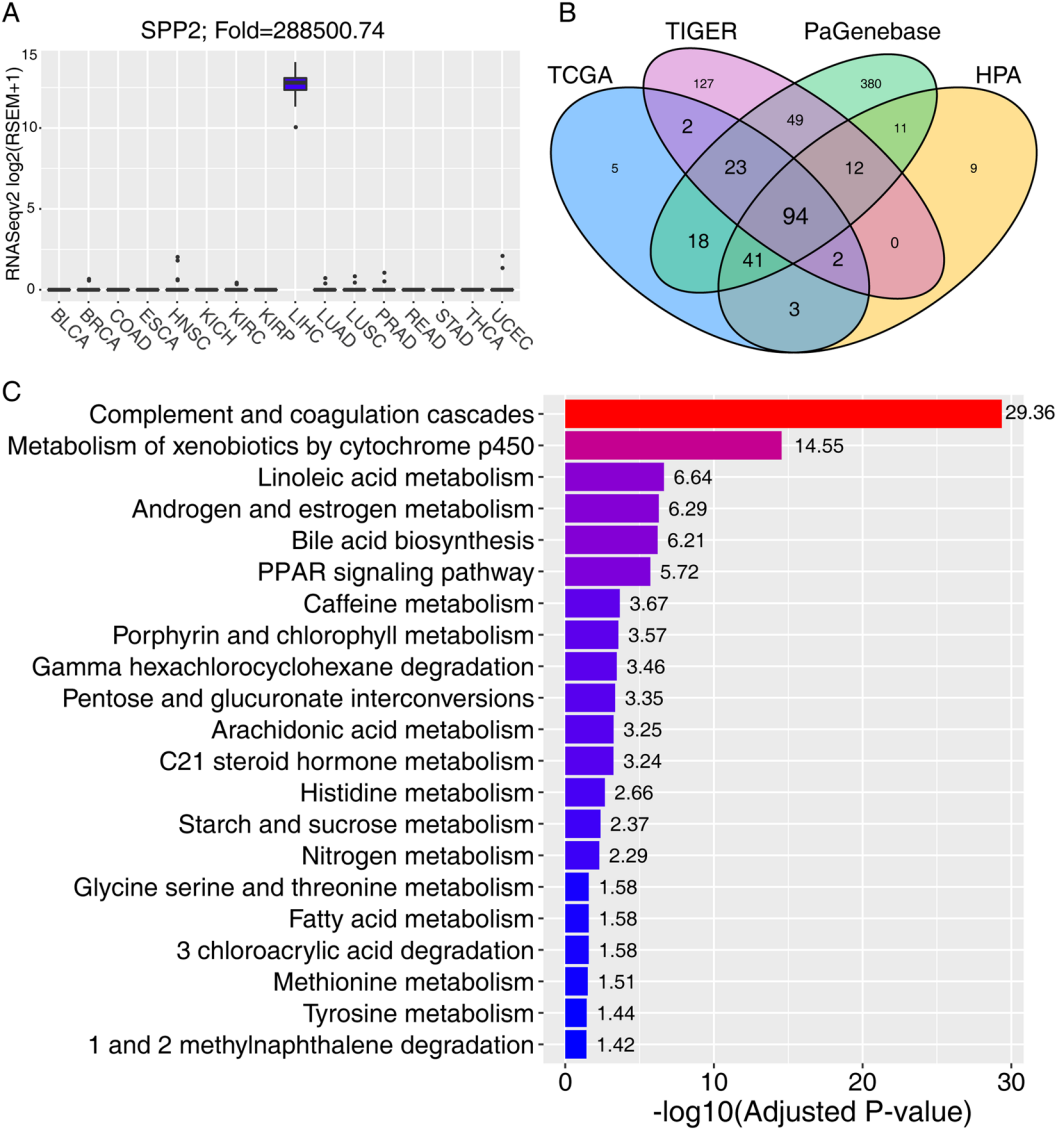
Validation of liver-enriched genes and KEGG analysis. (**A**) An example of liver-enriched genes. SPP2 was exclusively expressed in the corresponding non-tumor tissues of HCC. (**B**) Four-set Venn diagram showing the overlap of the liver-enriched genes derived from the TCGA and three other databases, including HPA, PaGenBase and TiGER. (**C**) Significantly enriched KEGG pathways of 188 liver-enriched genes. −log10(adjusted p-value) was annotated on each bar of the KEGG pathway.

We focused on LEGs in subsequent analyses because of the essential role of the liver in TEGs. One example of an LEG is SPP2 (secreted phosphoprotein 2), which is exclusively expressed in the corresponding non-tumor tissues of HCC (Fig. 3A). Furthermore, to validate the screening efficiency and accuracy, we queried public accessible databases for gene expression profiles, including the Human Protein Atlas (HPA)^17^, the Pattern Gene Database (PaGenBase)^18^ and the Tissue-specific Gene Expression and Regulation (TiGER)^19^ databases, from which we obtained 172, 628 and 309 LEGs, respectively. LEGs generated from our algorithm, and the HPA, PaGenBase, and TiGER databases overlapped significantly. Among the 188 LEGs obtained from our algorithm, a total of 183 (183/188, 97.3%) genes were identified in two or more than two databases, and 94 (94/188, 50%) were identified in all three databases (Fig. 3B).

To explore the characteristics of the 188 LEGs, KEGG pathway enrichment analyses were conducted with Enrichr, and 21 significantly enriched pathways were identified (Fig. 3C). Consistent with the metabolic functions of the liver, most LEGs were distributed in metabolic pathways, including xenobiotic metabolism, linoleic acid metabolism, sex hormone metabolism and bile acid biosynthesis. These results suggest that LEGs represent the biological functions of the liver.

### LEGs have similar expression patterns in HCC tissues

**Figure 4:**
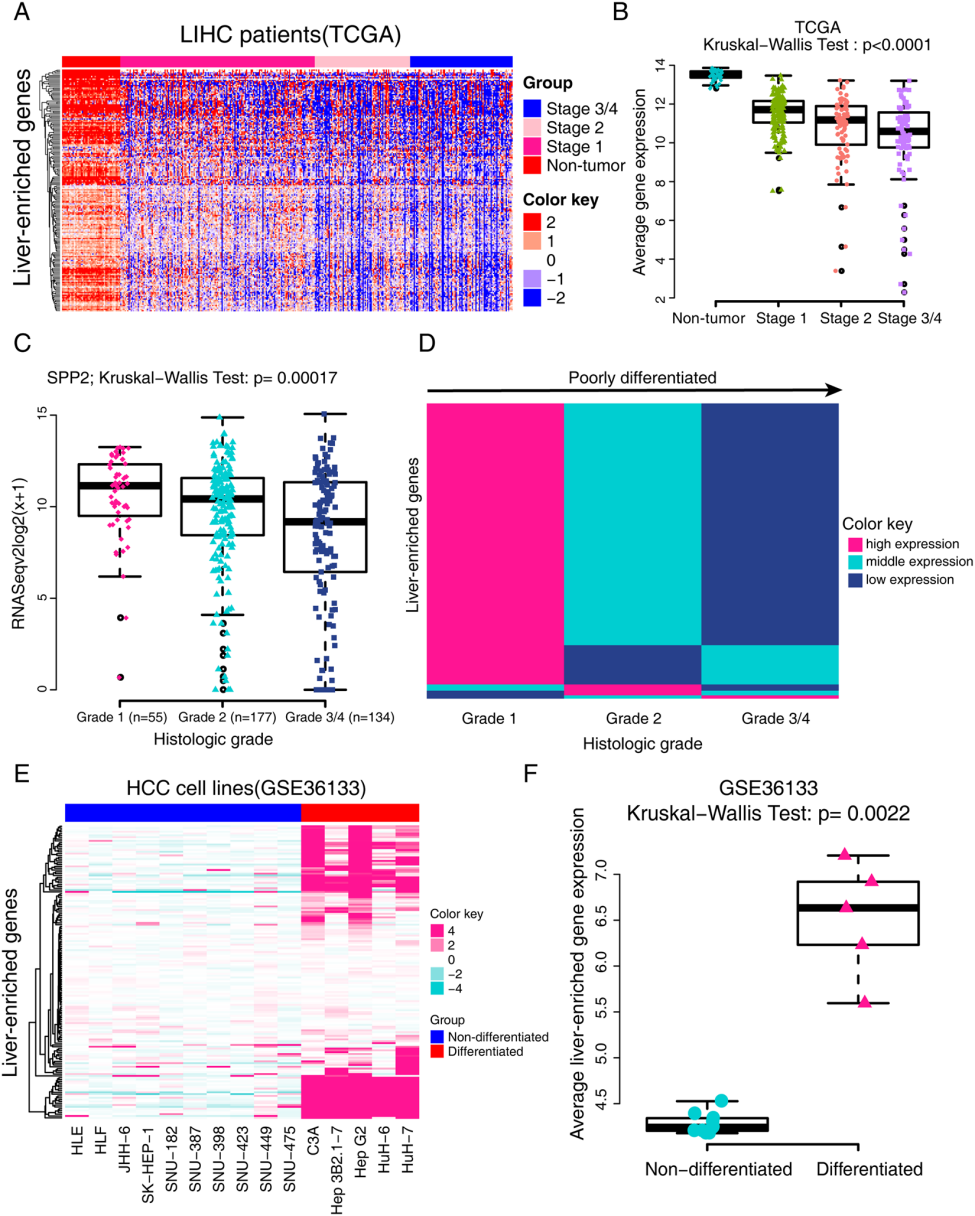
Expression pattern of liver-enriched genes and its relationship with tumor dedifferentiation. (**A**) Heatmap displaying the expression pattern of liver-enriched genes in non-tumor tissues and HCC tissues with different pathological stages in the TCGA dataset. Red indicates high expression and blue indicates low expression. (**B**) Quantitative analysis of the expression pattern of liver-enriched genes in Fig. 4A. RSEM values were Log2 transformed, and the mean values of the liver-enriched genes in every sample were calculated. Then the Kruskal-Wallis test was used to compare differences among the four groups. (**C**) SPP2 was a typical example showing the expression level of liver-enriched genes in different histological grades. SPP2 expression decreased gradually with the progression of the tumor grade. (**D**) Heatmap showing the expression levels of 188 liver-enriched genes in different histological grades. Most liver-enriched genes displayed gradually reduced RNA expression correlated with the augment of tumor grade. (**E**) Comparison of mRNA expression profiles of liver-enriched genes in differentiated and non-differentiated HCC cell lines in GSE36133. Ten were non-differentiated HCC cell lines (HLE, HLF, JHH-6, SK-HEP-1, SNU-182, SNU-387, SNU-398, SNU-423, SNU-449 and SNU-475) and five were differentiated cell lines (C3A, Hep3B2.1-7, HepG2, HuH-6 and HuH-7). (**F**) Quantitative analysis of the expression pattern of liver-enriched genes in the differentiated and non-differentiated HCC cell lines.

Then we investigated the expression profiles of LEGs in HCC. The heatmap shown in Fig. 4A illustrates that most LEGs were downregulated in tumor tissues in the TCGA dataset. The average gene expression of tumors was significantly less than that of non-tumor tissues, and a more advanced pathological stage accompanied less average expression of LEGs (Fig. 4B). We also analyzed the expression pattern of LEGs with microarray datasets from GEO. Heatmap and statistical analysis validated the downregulation of LEGs in both GSE14520 (Supplementary Fig. S1A,B) and GSE54236 (Supplementary Fig. S1C,D). Furthermore, most LEGs were positively correlated with each other, which indicates a similar expression pattern of LEGs in HCC (Supplementary Fig. S1E).

### Downregulation of LEGs is a sign of dedifferentiation

When we evaluated the correlation between histological grade and gene expression, we observed that the expression of some genes was closely correlated with histological grade. For example, SPP2 expression significantly decreased with advancing tumor grade (Fig. 4C). Then we analyzed the association between the expression pattern of all LEGs and tumor grade. Most (82%, 154/188) LEGs downregulated as the histological grade progressed (Fig. 4D).

To better understand the association between LEGs and dedifferentiation, we further analyzed the expression of LEGs in cell lines from GSE36133^20^. The heatmap revealed a clear boundary between differentiated and non-differentiated HCC cell lines (Fig. 4E). Average expression of LEGs in the differentiated cell lines was significantly higher than that in the non-differentiated HCC cell lines (p = 0.0022, Fig. 4F). Overall, these results indicate that downregulation of LEGs is a sign of dedifferentiation in patients with HCC and the HCC cell lines.

### LEGs are hypermethylated in HCC tissues

**Figure 5:**
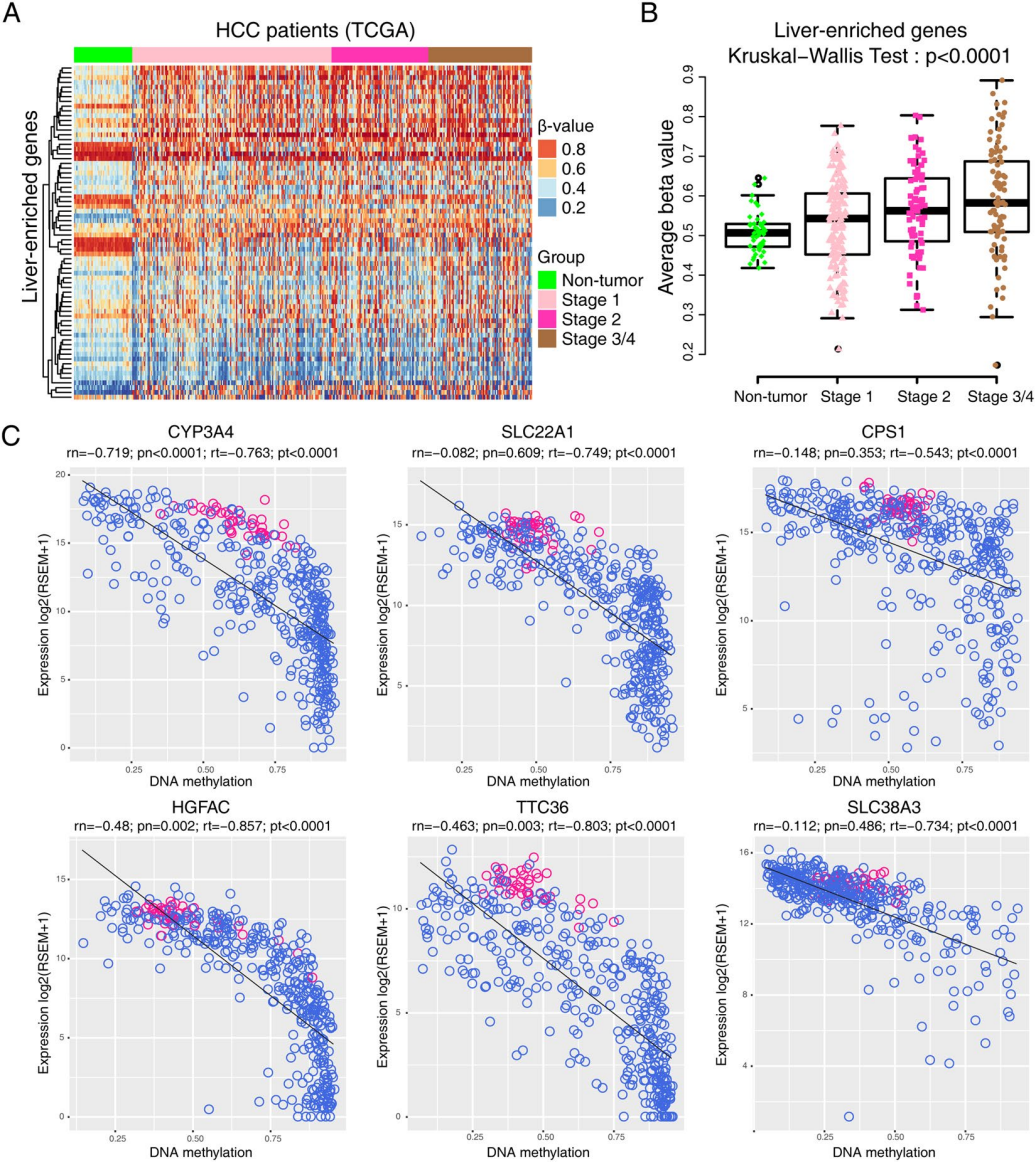
Methylation patterns of liver-enriched genes. (**A**) Heatmap showing the methylation patterns of liver-enriched genes in non-tumor tissues and HCC of different pathological stages. The beta values were used to draw the heatmap. (**B**) Average methylation levels of liver-enriched genes in non-tumor and tumor tissues. (**C**) Scatterplots of representative liver-enriched genes shown to be hypermethylated in HCC, where gene RNA expression (y axis) is plotted against relative hypermethylation (x axis). Blue dots are results from tumor samples, and pink dots are results from normal tissues. Abbreviations in figures: rn, Spearman r value of non-tumor tissue; pn, p-value of non-tumor tissue; rt, Spearman r value of tumor tissue; pt, p-value of tumor tissue.

Next, we explored why LEGs were downregulated in tumors. Methylation alters the readability of DNA and results in a change of mRNA transcription in HCC^21^. We compared the DNA methylation profiles of LEGs in normal tissue and HCC. A heatmap revealed hypermethylation of LEGs in HCC at different pathological stages (Fig. 5A). DNA methylation of LEGs was significantly higher in advanced pathological stages of HCC and was lowest in adjacent non-tumor liver tissues (Fig. 5B). Six representative genes whose expression was significantly inverse correlated with methylation level are shown in Fig. 5C. These genes displayed significantly reduced RNA expression correlated with hypermethylation. Of the six representative genes, CYP3A4^22^, SLC22A1^23^, CPS1^24^ were previously reported to be regulated by methylation. Although HGFAC, TTC36, and SLC38A3 are highly hypermethylated and downregulated genes, no previous study has reported their methylation. Hypermethylation-mediated downregulation might be a possible mechanism underlying the expression pattern of LEGs in HCC.

### The LEG set is associated with the prognosis of patients with HCC

**Figure 6:**
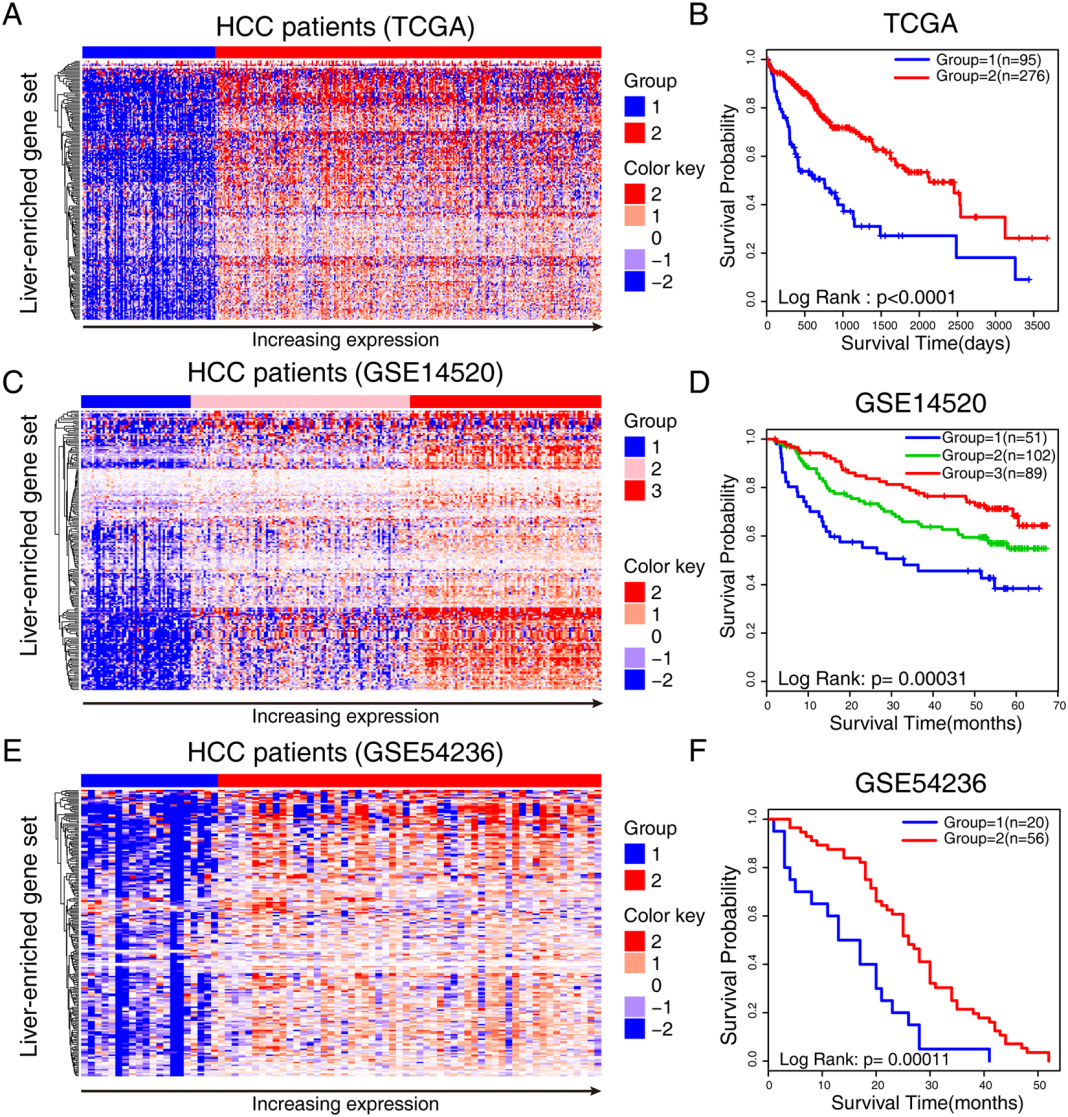
Liver-enriched gene set is associated with the prognosis of patients with HCC. Heatmap shows the unsupervised consensus clustering result of the liver-enriched genes in the (**A**) TCGA, (**C**) GSE14520, and (**E**) GSE54236. The expression level of liver-enriched genes increased from blue to red color. (**B**) Survival analysis of the groups generated from consensus clustering with the (**B**) TCGA, (**D**) GSE14520, and (**F**) GSE54236 datasets.

Given that more than half of the LEGs were prognostic (Fig. 2C), we established a gene set with 188 LEGs and assessed its prognostic capability. Consensus clustering was employed to cluster patients for its advantages in assessing the stability and robustness of the discovered clusters particularly in gene expression data^25^. We first performed consensus clustering with the TCGA dataset and generated two stable clusters (Supplementary Fig. S2A,B). Strong differences in the expression patterns of the LEGs were observed between the two groups (Fig. 6A, Supplementary Fig. S2C). The cluster with high LEG expression had prolonged overall survival (Fig. 6B). Next, we validated the prognostic ability of the gene set with microarray data. Consensus clustering identified three stable and robust clusters using the GSE14520 dataset (Supplementary Fig. S2D,E). Strong differences in survival probabilities were observed among the three subtypes. The expression of LEGs increased from groups 1 to 3 (Fig. 6C, Supplementary Fig. S2F), and group 3 had the highest LEG expression and the best prognosis, whereas group 1 with the lowest LEG expression had the worst prognosis (Fig. 6D). Similarly, consensus clustering recognized two clusters in GSE54236 (Supplementary Fig. S2G,H) with distinct LEG expression (Fig. 6E, Supplementary Fig. S2I), and higher expression of LEGs was associated with a good clinical outcome (Fig. 6F). Taken together, these results highlight the favourable prognostic effects of the LEG set and provide important insights into the potential protective role of LEGs.

### Correlations between mean expression distance and HCC biological behaviors

**Figure 7:**
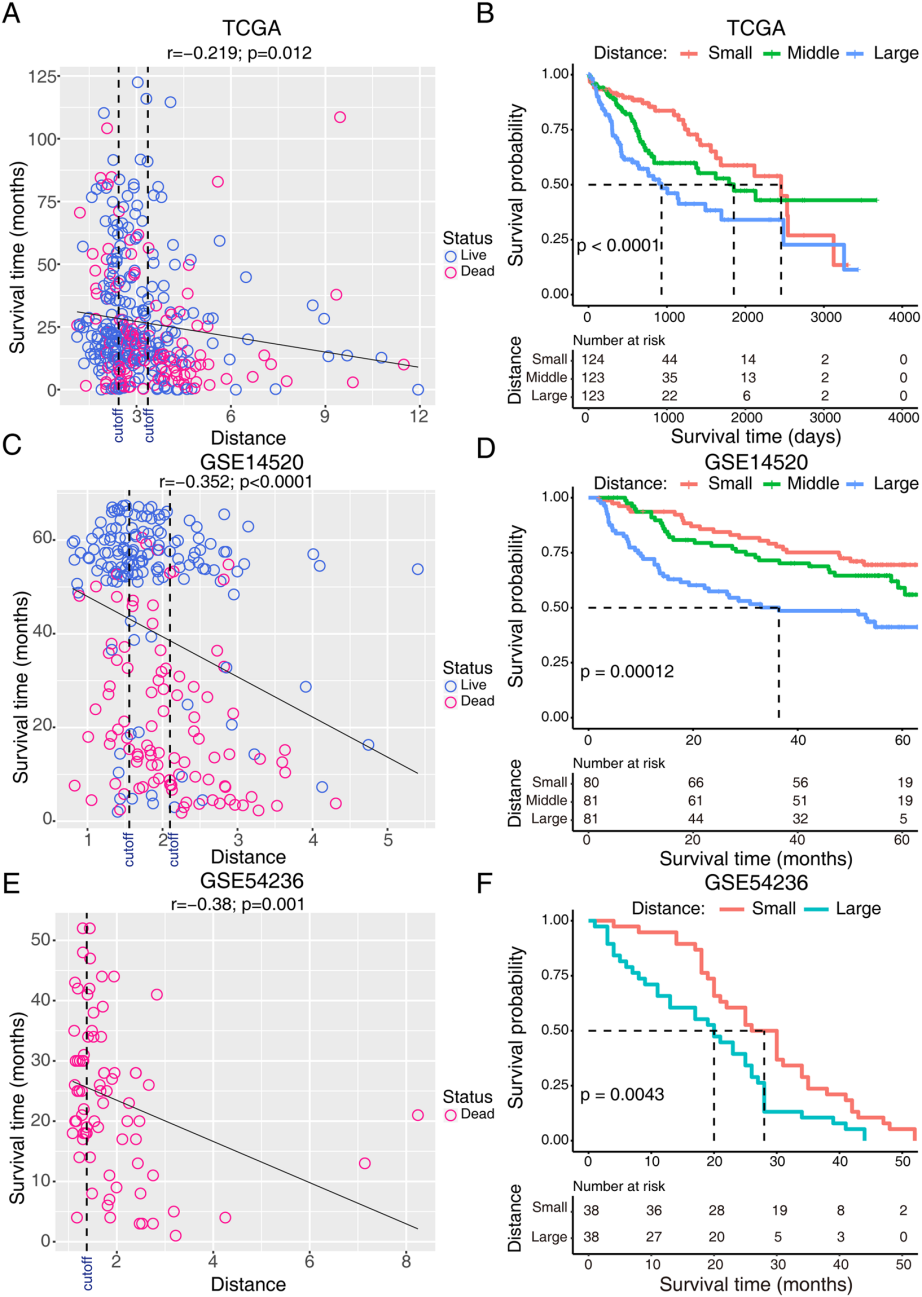
Correlations between mean expression distance and the prognosis of patients with HCC. Scatterplots of survival time and mean expression distance in the (**A**) TCGA, (**C**) GSE14520, and (**E**) GSE54236, where survival time (y axis) is plotted against mean expression distance (x axis). Blue and pink represented patients with live or dead survival status, respectively. Black dashed line represents the cut-off value for dividing patients into different groups. The survival curves of different groups in the (**B**) TCGA, (**D**) GSE14520, and (**F**) GSE54236 datasets.

We noticed that LEGs were downregulated in HCC, and the degree of reduced expression was associated with the clinical outcome of patients with HCC in which the most downregulated subgroup had the poorest prognosis. This phenomenon raised the question as to whether the distance between tumors and normal tissues could be used as an indicator to measure the biological behavior of tumors and evaluate the prognosis of patients with HCC. We calculated the mean expression distance of the LEGs between tumors and normal tissues. The patients with HCC in the TCGA dataset were equally divided into three groups (small distance, middle distance, and large distance) by two cut-off values according to the mean expression distance from normal tissues (Fig. 7A). Survival time was negatively correlated with the mean expression distance (Fig. 7A). Patients with the largest expression distance exhibited the shortest survival time, whereas patients with the smallest expression distance showed a substantial advantage in overall survival. Patients with middle expression distances exhibited median survival times (Fig. 7B). Similar results were observed in the two aforementioned external cohorts. Survival time was inversely correlated with distance in GSE14520, and most live patients were distributed in groups with small or middle expression distances (Fig. 7C). Patients with a large expression distance had a significantly poorer prognosis than patients with small and middle distances (Fig. 7D). Similarly, a large expression distance was associated with a poorer prognosis in GSE54236 (Fig. 7E,F).

To assess whether the expression distance of LEGs predicts the prognosis of patients with HCC, we regarded the distance as risk scores and used the C-index, which measures the fraction of all pairs of individuals whose predicted survival times are ordered correctly^26^, and the Brier score, which calculates the error of the model fit on survival data^27^. On average, the TCGA and GEO datasets generated a high C-index and a low Brier score (Table 1, Supplementary Fig. S3A–C). We assessed the prognostic accuracy with time-dependent receiver operating characteristic (ROC) curve analysis at various follow-up times (Supplementary Fig. S3D–F). The area under the curve(AUC) at different cut-off times indicated an acceptable predictive accuracy. These results demonstrate the robustness of the expression distance of LEGs for predicting survival outcomes.

**Table 1:** Performance of the model using mean expression distance as the risk score in the TCGA cohort and GEO datasets.

Dataset	C-index	95%CI	p-value	Brier score
TCGA	0.637	0.584–0.692	5.83E-07	0.202
GSE14520	0.631	0.573–0.689	8.84E-06	0.181
GSE54236	0.631	0.563–0.700	1.64E-04	0.131

In addition, we evaluated the correlation between mean expression distance and clinical variables (Table 2). A large expression distance was associated with a higher alpha fetoprotein (AFP) level, tumor weight, advanced histological and pathological stages, and vascular invasion. Interestingly, we also determined that patients with a large expression distance tended to be females, younger age and low body weight, which suggests a correlation between the tumor and its host. Taken together, these results indicate that the divergence between tumors and normal tissues predicted the prognosis of patients with HCC and possibly affected the biological behaviors of the tumors.

**Table 2:** Relationship between mean expression distance and clinical characteristics of patients with HCC.

Characteristics	Small distance	Middle distance	Large distance	p-value
Age				0.002^*^
Mean	62.85	59.59	55.86	
SD	10.78	13.46	15.15	
Gender				0.004^*^
Male	97 (38.8%)	80 (32.0%)	73 (29.2%)	
Female	27 (22.3%)	43 (35.5%)	51 (42.1%)	
Patient weight				p < 0.0001^**^
Mean	78.81	73.3	66.14	
SD	19.42	21.5	14.63	
AFP				p < 0.0001^**^
<200 µg/L	91 (45.3%)	67 (33.3%)	43 (21.4%)	
≥200 µg/L	7 (9.1%)	30 (39.0%)	40 (51.9%)	
Child-Pugh grade				0.334
a	84 (38.7%)	76 (35.0%)	57 (26.3%)	
b/c	5 (22.7%)	10 (45.5%)	7 (31.8%)	
Tumor weight				p < 0.0001^**^
Mean	249.54	242.31	427.46	
SD	323.51	320.06	587.43	
Vital status				0.006^*^
Alive	93 (38.6%)	79 (32.8%)	69 (28.6%)	
Dead	31 (23.8%)	44 (33.8%)	55 (42.3%)	
Histological grade				p < 0.0001^**^
G1	24 (43.6%)	21 (38.2%)	10 (18.2%)	
G2	80 (45.2%)	51 (28.8%)	46 (26.0%)	
G3/4	19 (14.2%)	48 (35.8%)	67 (50%)	
Pathological stage				p < 0.0001^**^
Stage I	76 (44.4%)	61 (35.7%)	34 (19.9%)	
Stage II	23 (26.7%)	27 (31.4%)	26 (28.9%)	
Stage III/IV	16 (17.8%)	26 (28.9%)	48 (53.3%)	
T stage				p < 0.0001^**^
T1	81 (44.8%)	65 (35.9%)	35 (19.3%)	
T2	24 (25.5%)	30 (31.9%)	40 (42.6%)	
T3/4	17 (18.3%)	27 (29.0%)	49 (52.7%)	
Inflammation				0.627
No	50 (42.7%)	35 (29.9%)	32 (27.4%)	
Mild	35 (35.4%)	28 (28.3%)	36 (36.4%)	
Severe	7 (38.9%)	8 (44.4%)	3 (16.7%)	
Vascular invasion				p < 0.0001^**^
No	86 (41.7%)	69 (33.5%)	51 (24.8%)	
Yes	24 (22.0%)	34 (31.2%)	51 (46.8%)	

All ordinal data were analyzed with nonparametric statistics as appropriate. Statistical significance is marked with the star symbol: *p < 0.01, **p < 0.0001.

## Discussion

TEGs are expressed at a much higher levels in a specific tissue types than in others, which is important for maintaining a tissue-specific biological function^3^. Here we identified TEGs from 12 tissue types with the RNA sequencing data, and obtained the largest gene number of genes from the liver. One well-known early study that is often cited in proteome research is that of Uhlen *et al*.^17^. They reported that the largest number of TEGs is found in the testes, followed by the brain and the liver. This inconsistency between that study and ours may be due to the limited tissue types analyzed in our study. To improve the statistical accuracy, we ruled out cancer types with less than 10 corresponding non-tumor tissues, and only 12 tissue types fully met the inclusion criteria. Several previous studies have identified TEGs with cut-off values of three-fold^12^, five-fold^28^ or 10-fold^29^. We raised the cut-off value to 100-fold to define a TEG, to ensure the specificity and to cover the shortage of limited tissue types. Fortunately, the efficiency of our algorithm in recognizing TEGs was validated, as shown in Fig. 3B. Because there was a significantly larger number of TEGs in the liver, and most LEGs were prognostic, we focused only on the characteristics of LEGs in this study.

HCC is the leading cause of cancer-related death worldwide and the clinical outcome of HCC patients is unsatisfactory^30,31^. Identifying prognostic factors helps improve the prognosis of patients with HCC. We have identified single prognostic genes in our previous studies including GLS2 and UPB1^6,7^, which are two genes mainly expressed in the liver. Consistent with the finding that hypermethylation might be responsible for the downregulation of LEGs, the GLS2 promoter is hypermethylated in tumor tissues^32–34^. In this study, we discovered that most LEGs are prognostic and established an LEG set that could be used to create clusters of patients with HCC and survival differences, but we could not evaluate the prognosis of individual patients or assess the predictive performance because the consensus class generated from consensus clustering was discrete. Nevertheless, this study helped predict the prognosis of patients with HCC, because we demonstrated that the distance measure was predictive and we identified many prognostic LEGs, most of which are reported to be secretory^17,29^. These results help point out a possible direction for screening HCC prognostic biomarkers, particularly those from serum.

Differentiation is a process in which a cell changes from one cell type to a more specialized type, thus developing the functions of their ultimate fate^35^. By contrast, dedifferentiation entails the loss of specific form or function of a mature cell but retaining the ability to proliferate, which is one hypotheses for the cellular origin of cancer^36,37^. Previous studies regarded the downregulation of TEGs as a sign of dedifferentiation^17,38^. Most liver-enriched proteins are metabolism-related or the products synthesized by the liver, such as complement and coagulation factors. Thus, LEGs represent the particular functions of the liver. The downregulation of LEGs indicates loss of normal liver functions. The low expression of LEGs was associated with an unfavorable clinical outcome (Fig. 5), which suggests that the farther the tumor tissue were from the normal liver, the poorer the patient prognosis. We calculated the mean expression distance from tumors to normal tissues and demonstrated that the dissimilarity between them was correlated with the prognosis of patients with HCC and the biological behaviors of the tumors. These findings will assist our understanding of the development and advancement of tumors.

The expression of LEGs decreased during tumorigenesis and cancer progression, as the expression profile of LEGs was negatively correlated with pathological stage. Hypermethylation and dedifferentiation might partly explain the downregulation of LEGs. However, further investigation and experimentation needs to be undertaken to uncover the underlying molecular mechanisms of the downregulation, decipher the functions of LEGs and further identify whether LEGs are passenger genes or driver genes in tumorigenesis. In addition, this study has raised the question of whether replacing the lost or damaged liver function by overexpressing LEGs in tumor cells would reverse the dedifferentiation process, restore the liver function and finally improve the prognosis of patients with HCC.

In conclusion, this study has established an LEG profile that might be helpful in evaluating the prognosis of patients with HCC, and demonstrated that LEGs were gradually downregulated with advancing pathological stage and dedifferentiation. Hypermethylation might be a possible mechanism underlying the decreased expression of LEGs. Moreover, we discovered that the degree of the dissimilarity between tumors and normal tissues predicts the prognosis of patients with HCC and is correlated with the biological behaviours of the tumors, which might contribute to expanding our understanding of cancer development. Further studies need to be carried out to elucidate the potential involvement of these LEGs in hepatocarcinogenesis and progression.

## Materials and Methods

### TCGA data sources

The RNA-seq and clinical data of different cancer categories (up to January 28, 2016) were retrieved from the TCGA database (http://gdac.broadinstitute.org/). Cancer types with more than 10 paired normal samples were included in the study. Thus, 12 tissue types and 16 cancer types met the requirement, consisting of 707 non-tumor samples and 7,091 tumor samples. The DNA methylation data were obtained from a Level 3 data “LIHC.meth.by_max_stddev.data” file, in which probes were matched to genes with the maximum standard deviation for each gene, and 10,015 genes were covered including 77 LEGs.

### Identification of tissue-enriched genes

To be identified as a tissue-enriched gene, the RNA-Seq by expectation-maximization (RSEM) value of the gene in relevant tissue was required to meet two criteria: 1) The mean RSEM value ranked first among all non-tumor tissues and was significantly different from second place; 2) the mean RSEM value of the relevant tissue was required to be 100 times greater than that of all remaining tissues.

### GEO data availability

GSE14520^39,40^ and GSE54236^41^ are two independent expression microarray HCC data sets with genome-wide level probes and complete information about the overall survival time and the survival status at the last follow-up^12^. Thus, these two datasets were downloaded from the Gene Expression Omnibus (GEO) database (http://www.ncbi.nlm.nih.gov/geo/). The survival time of patients with HCC in the microarrays was retrieved from ArrayExpress (http://www.ebi.ac.uk/arrayexpress/). The GSE14520 dataset consisted of 242 patients with primary HCC, and the GSE54236 dataset included 80 patients with HCC. The GSE36133 dataset^20^ contained the sequencing expression data from the Cancer Cell Line Encyclopaedia (CCLE) from nearly 1,000 human cancer cell lines of 36 cancer types including 26 HCC cell lines, 10 of which were non-differentiated HCC cell lines (HLE, HLF, JHH-6, SK-HEP-1, SNU-182, SNU-387, SNU-398, SNU-423, SNU-449, and SNU-475) and five cell lines were differentiated cell lines (C3A, Hep 3B2.1-7, HepG2, HuH-6 and HuH-7)^42^.

### Gene expression profile databases

The PaGenBase (http://bioinf.xmu.edu.cn/PaGenBase/) is a free database that provides information of pattern genes (specific genes, selective genes, housekeeping genes and repressed genes) of 11 model organisms^18^. The TiGER (http://bioinfo.wilmer.jhu.edu/tiger/) is a database containing tissue-specific gene expression profiles^19^. The HPA is a tissue-based map of the human proteome from analyses of 32 tissues and 47 cell lines, with gene expression data at both the RNA and protein levels^17^. These three databases identified 628, 309 and 172 LEGs, respectively.

### Consensus clustering analyses

Data were log2 transformed and median centered for normalization before clustering analysis. We utilized the R package ConsensusClusterPlus^28^ to identify robust clusters. To perform consensus clustering, we used K-mean approach with Euclidean distance. The procedure was run with 1,000 iterations with max K = 6, and a sub-sampling ratio of 0.8. Heatmaps were generated using Bioconductor package complexHeatmap^43^.

### Enrichment analyses

Biological pathways analyses based on KEGG pathway database were performed with Enrichr (http://amp.pharm.mssm.edu/Enrichr/)^44^. Pathways with adjusted p-value < 0.05 were considered to be significantly enriched.

### Survival analyses

The relationship between survival and gene expression was analyzed using the “survfit” function in the R survival package. The significance of overall survival was determined by the Log-rank test. Survival analyses were performed in R (version 3.4.0), and the survival curve was generated by the R survival package or the survminer package.

### Calculation of the expression distance between tumors and corresponding normal tissues and evaluation of the accuracy of the survival prediction

The Euclidean distance was calculated to measure the divergence between tumors and normal tissues with a formula modified from Hu *et al*.^45^. Mean expression distance (med) was calculated as follows:

$$me{d}_{j}=\frac{1}{n}\sum _{i=1}^{n}\sqrt{\frac{1}{p}(\sum _{k=1}^{p}{({\mathrm{log}}_{2}{y}_{ij}-{\mathrm{log}}_{2}{x}_{ik})}^{{\rm{2}}})}$$

where ${x}_{ij}$ and ${y}_{ij}$ are the expression of gene $i$ over two expression profiles with $p$ normal and $q$ tumor samples $({x}_{1},{x}_{2},\mathrm{....},{x}_{p}),({y}_{1},{y}_{2},\mathrm{....},{y}_{q})$, and $n$ is the number of LEGs. The expression value was log2 transformed. The mean expression distance of every tumor tissues was calculated $(me{d}_{1},me{d}_{2},\mathrm{...},me{d}_{j}\mathrm{....}me{d}_{q})$. Patients were sorted according to increasing $med$ and divided equally into two or three groups.

The mean expression distance was considered as “risk score”, and the Concordance index(C-index)^26^, Brier score^27^ and time-dependent receiver operating characteristic (ROC) curve were used to evaluate the accuracy of survival prediction of the mean expression distance. The C-index was calculated using the function concordance.index and the Brier score was calculated with the function sbrier.score2proba in the R survcomp package^46^. The time-dependent ROC curves were generated from the function survivalROC in the R survivalROC package^47^.

### Availability of data and materials

All data and materials are available upon request.

## Electronic supplementary material

Table S1

supplementary figure
