# Supplementary material for: Liver-enriched Genes are Associated with the Prognosis of Patients with Hepatocellular Carcinoma
Source: Sci Rep. 2018 Jul 25;8:11197. doi: 10.1038/s41598-018-29237-5 (PMC6060164; doi:10.1038/s41598-018-29237-5)
Supplement: Supplementary file 2 — supplementary figure [file 41598_2018_29237_MOESM2_ESM.pdf]

Liver-enriched Genes are Associated with the Prognosis of Patients with Hepatocellular Carcinoma

Binghua Li[1, *], Tiancheng Xu[1, *], Chaohui Liu[2], Gang Meng[2], Yuchen Sun[1], Liyuan Qian[1], Shaohe Wang[1], Jiwu Wei[2], Decai Yu[1], Yitao Ding[1]

[1] Department of Hepatobiliary Surgery, the Affiliated Drum Tower Hospital, Medical School of Nanjing University, Nanjing, 210093, China
[2]Jiangsu Key Laboratory of Molecular Medicine, Medical School of Nanjing University, Nanjing, 210093, China

*These authors contributed equally to this study. Correspondence and requests for materials should be addressed to D.C.Y. (email: dryudecai@qq.com) or Y.T.D. (email: yitao_ding@163.com)

Figure S1. Expression pattern of liver-enriched genes. Heatmap showing the expression patterns of liver-enriched genes in non-tumor tissue and tumor tissue in (A) GSE14520 and (C) GSE54236. Red indicates higher expression and blue indicates lower expression. Quantitative analysis of the expression patterns of liver-enriched genes in non-tumor and tumor tissues in (B) GSE14520 and (D) GSE54236. (E) Heatmap showing the internal correlation of the 188 liver-enriched genes based on Spearman's correlation coefficient. The color in the heatmap represents the Spearman's correlation coefficient. Red indicates positive correlation, and blue indicates negative correlation.

Figure S2. Consensus clustering of liver-enriched genes in the TCGA and GEO datasets. Consensus clustering matrix of liver-enriched genes for k=2 to k=5 of (A) TCGA, (D) GSE14520, and (G) GSE54236. Consensus clustering CDF for k=2 to k=6 of (B) TCGA, (E) GSE14520, and (H) GSE54236. (C) Mean expression values for liver-enriched genes of the patients in the different groups in (C) TCGA, (F) GSE14520, and (I) GSE54236.

Figure S3. Time-dependent Brier score curves and ROC curves according to mean expression distance in the TCGA and GEO datasets. The time-dependent Brier score curve of (A) the TCGA LIHC cohort (B) the GSE14520 dataset, and (C) the GSE54236 dataset. The time-dependent ROC curve of (D) the TCGA LIHC cohort (E) the GSE14520 dataset, and (F) the GSE54236 dataset.

Table S1. The tissue-enriched gene list of 12 tissue types.

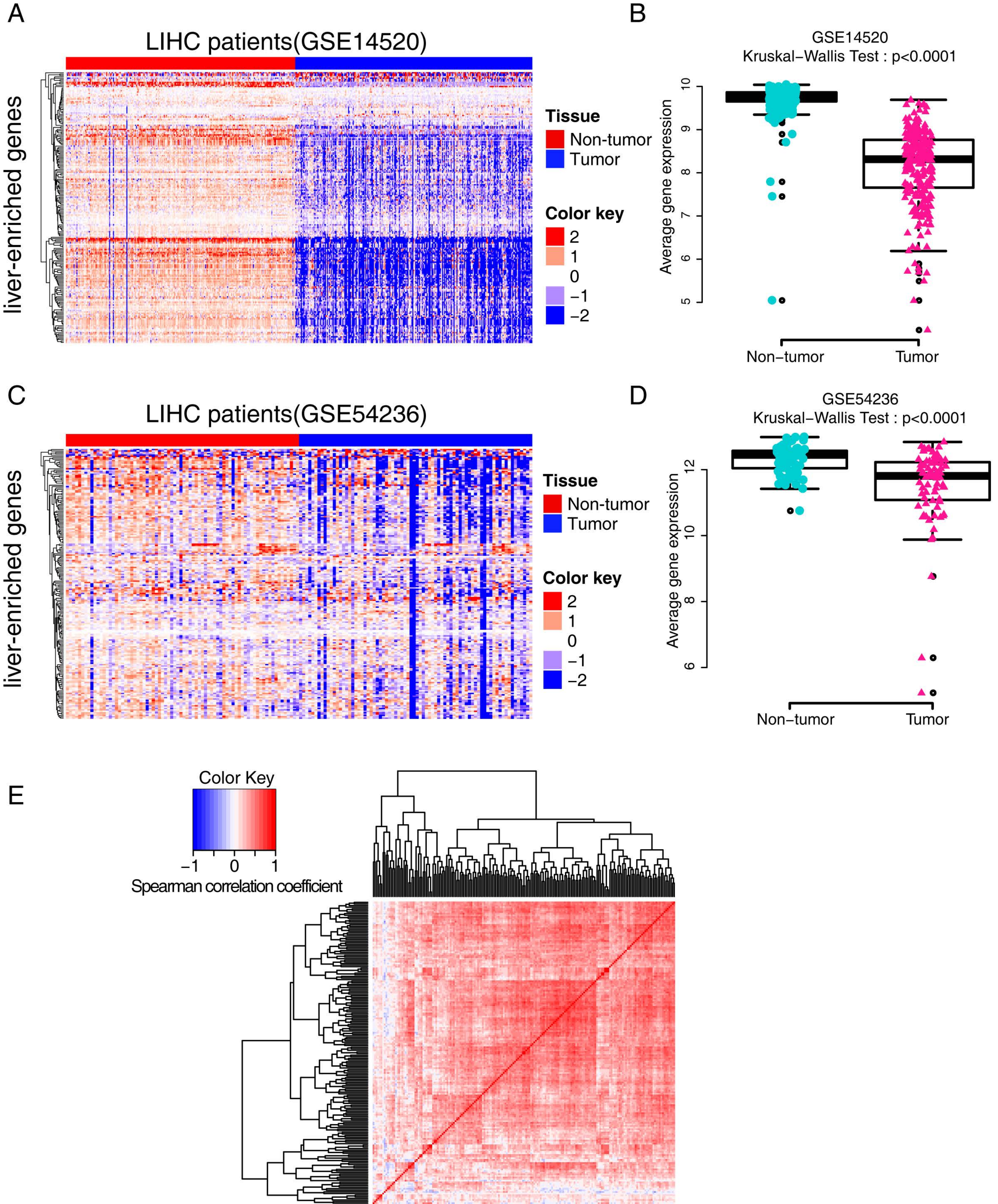
A
LIHC patients(GSE14520)
liver-enriched genes
Tissue
Non-tumor
Tumor
Color key
2
1
0
−1
−2
B
GSE14520
Kruskal−Wallis Test : p<0.0001
Average gene expression
5
6
7
8
9
10
Non−tumor
Tumor
C
LIHC patients(GSE54236)
liver-enriched genes
Tissue
Non-tumor
Tumor
Color key
2
1
0
−1
−2
D
GSE54236
Kruskal−Wallis Test : p<0.0001
Average gene expression
6
8
10
12
Non−tumor
Tumor
E
Color Key
−1
0
1
Spearman correlation coefficient

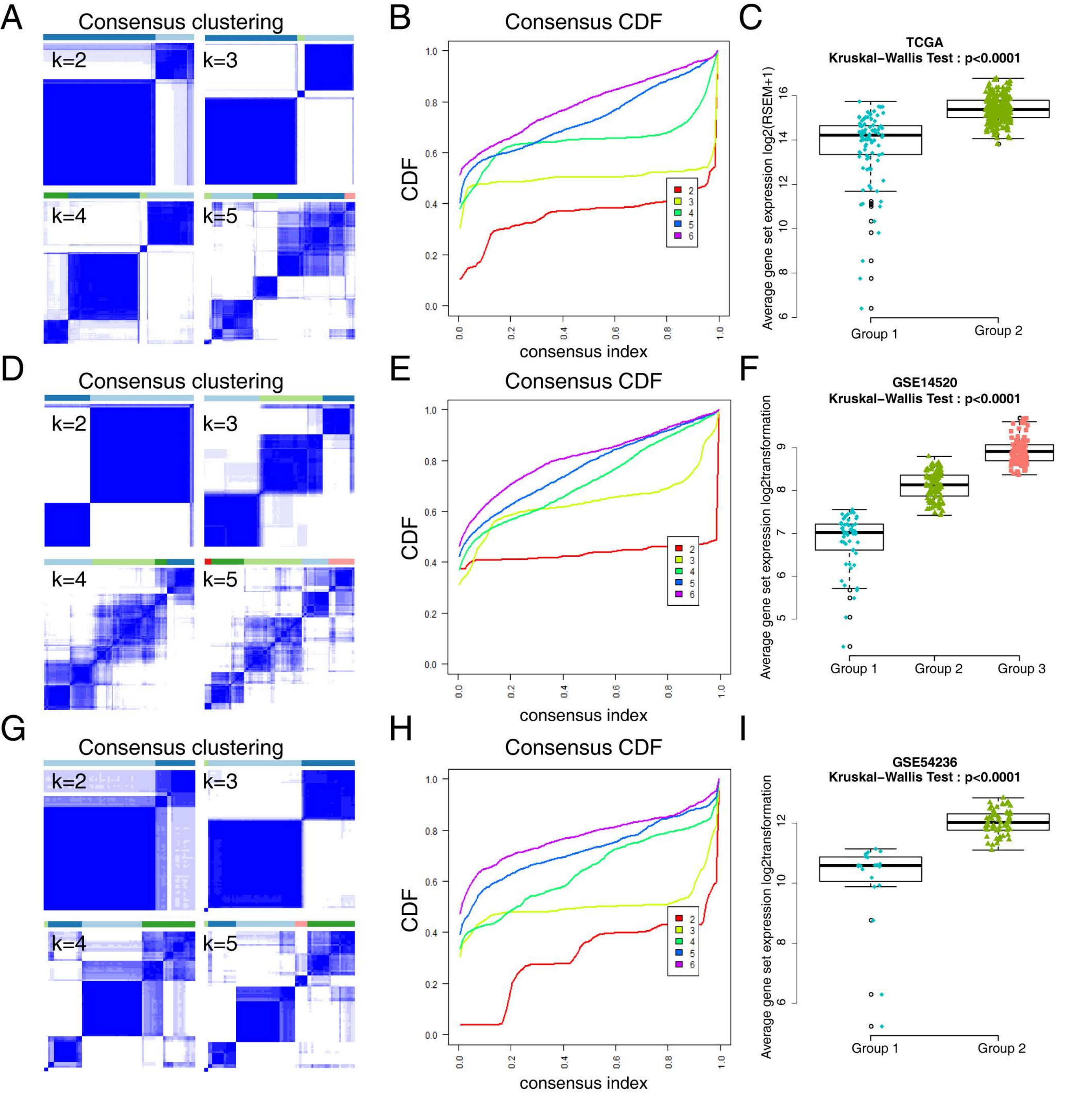
A
Consensus clustering
k=2
k=3
k=4
k=5
B
Consensus CDF
CDF
consensus index
2
3
4
5
6
C
TCGA
Kruskal-Wallis Test : p<0.0001
Average gene set expression log2(RSEM+1)
Group 1
Group 2
D
Consensus clustering
k=2
k=3
k=4
k=5
E
Consensus CDF
CDF
consensus index
F
GSE14520
Kruskal-Wallis Test : p<0.0001
Average gene set expression log2transformation
Group 1
Group 2
Group 3
G
Consensus clustering
k=2
k=3
k=4
k=5
H
Consensus CDF
CDF
consensus index
I
GSE54236
Kruskal-Wallis Test : p<0.0001
Average gene set expression log2transformation
Group 1
Group 2

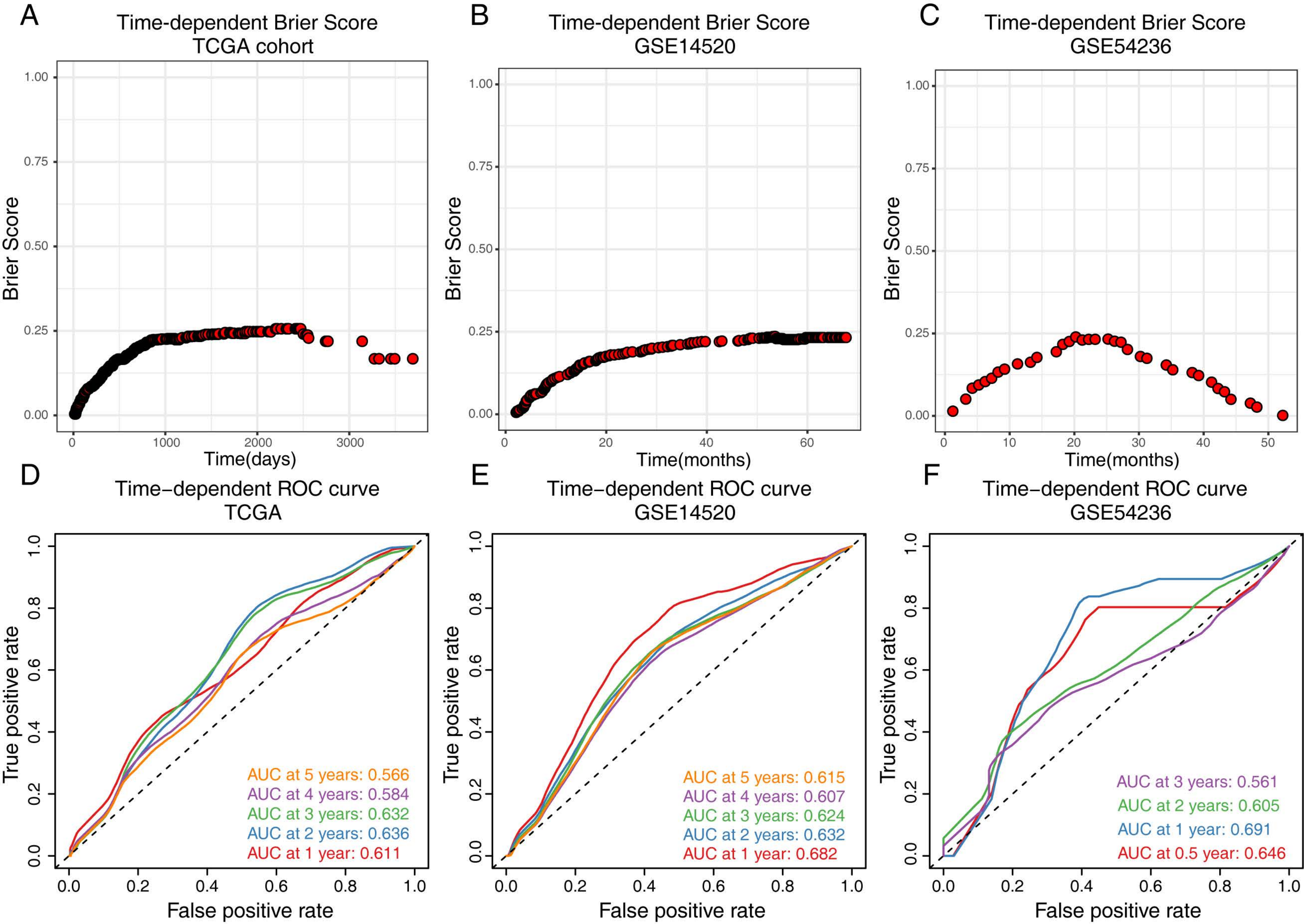
A
Time-dependent Brier Score
TCGA cohort
Brier Score
Time(days)
B
Time-dependent Brier Score
GSE14520
Brier Score
Time(months)
C
Time-dependent Brier Score
GSE54236
Brier Score
Time(months)
D
Time−dependent ROC curve
TCGA
True positive rate
False positive rate
AUC at 5 years: 0.566
AUC at 4 years: 0.584
AUC at 3 years: 0.632
AUC at 2 years: 0.636
AUC at 1 year: 0.611
E
Time−dependent ROC curve
GSE14520
True positive rate
False positive rate
AUC at 5 years: 0.615
AUC at 4 years: 0.607
AUC at 3 years: 0.624
AUC at 2 years: 0.632
AUC at 1 year: 0.682
F
Time−dependent ROC curve
GSE54236
True positive rate
False positive rate
AUC at 3 years: 0.561
AUC at 2 years: 0.605
AUC at 1 year: 0.691
AUC at 0.5 year: 0.646
